# Novel Insights Into the Mechanism of GVHD-Induced Tissue Damage

**DOI:** 10.3389/fimmu.2021.713631

**Published:** 2021-08-27

**Authors:** Takahide Ara, Daigo Hashimoto

**Affiliations:** Department of Hematology, Hokkaido University Faculty of Medicine, Sapporo, Japan

**Keywords:** allogeneic hematopoietic stem cell transplantation, GVHD, graft-versus-host disease, intestinal stem cells, tissue stem cells, microbiota, Paneth cell, goblet cell

## Abstract

Prophylaxis for and treatment of graft-versus-host disease (GVHD) are essential for successful allogeneic hematopoietic stem cell transplantation (allo-SCT) and mainly consist of immunosuppressants such as calcineurin inhibitors. However, profound immunosuppression can lead to tumor relapse and infectious complications, which emphasizes the necessity of developing novel management strategies for GVHD. Emerging evidence has revealed that tissue-specific mechanisms maintaining tissue homeostasis and promoting tissue tolerance to combat GVHD are damaged after allo-SCT, resulting in exacerbation and treatment refractoriness of GVHD. In the gastrointestinal tract, epithelial regeneration derived from intestinal stem cells (ISCs), a microenvironment that maintains healthy gut microbiota, and physical and chemical mucosal barrier functions against pathogens are damaged by conditioning regimens and/or GVHD. The administration of growth factors for cells that maintain intestinal homeostasis, such as interleukin-22 (IL-22) for ISCs, R-spondin 1 (R-Spo1) for ISCs and Paneth cells, and interleukin-25 (IL-25) for goblet cells, mitigates murine GVHD. In this review, we summarize recent advances in the understanding of GVHD-induced tissue damage and emerging strategies for the management of GVHD.

## Introduction

Mature epithelial cells in the gut, skin, and liver have long been recognized as the primary target of acute graft-versus-host disease (GVHD) after allogeneic hematopoietic stem cell transplantation (allo-SCT). Mature epithelial cells in the gut are composed of functionally distinct populations, including enterocytes, Paneth cells, goblet cells, tuft cells, and enteroendocrine cells. Each of these epithelial populations contributes to the maintenance of tissue homeostasis (**Table 1**). Thus, injury of these epithelial cells results in alteration of the tissue microenvironment and disruption of tissue homeostasis, potentially amplifying GVHD-induced tissue damage. Furthermore, emerging evidence indicates that adult tissue stem cells are primarily targeted by GVHD, which decreases tissue resilience in GVHD target organs (5, 7, 19). Here, we review recent advances in the understanding the cellular and molecular mechanisms of GVHD-induced tissue damage and disruption of the tissue microenvironment. This review mainly focuses on gastrointestinal GVHD, while recent findings on the injury of tissue stem cells in the other organs are also summarized.

**Table 1:** Intestinal cells that maintain intestinal homeostasis.

Cell Type	Location	Function	Mouse GVHD	Human GVHD	References
DCS cell	LI	Secrete ISC growth factors such as EGF and NOTCH ligands	Unknown	Unknown	Sasaki et al. (1), PMID: 27573849
Goblet Cell	SI/LI	Maintain the mucus layers by mucin production	↓	↓	Ara et al. (2), PMID: 32611682
ILC2	SI/LI	Secrete goblet cell growth factors such as IL-4/IL-13 in response to IL-33 and IL-25	↓ ^a)^	↓ ^a),b)^	Bruce et al. (3), PMID: 28375154 Munneke et al. (4), PMID: 24855210
ILC3	SI/LI	Secrete a ISC growth factor, IL-22	↓	Unknown	Hanash et al. (5), PMID: 22921121 Lindemans et al. (6), PMID: 26649819 Munneke et al. (4), PMID: 24855210
ISC	SI/LI	Differentiate into all types of intestinal epithelial cells	↓	↓	Takashima et al. (7), PMID: 21282378 Takashima et al. (8), PMID: 31811055
L Cell	SI/LI	Secrete a ISC growth factor, GLP-2	↓	↓	Norona et al. (9), PMID: 32542357
LEC	SI	Secrete a ISC growth factor, R-Spondin 3	↓	Unknown	Ogasawara et al. (10), 30013036
MRISC	LI	Secrete a ISC growth factor, R-Spondin 1 (Production of R-Spondin1 is enhanced in response to gut injury).	Unknown	Unknown	Wu et al. (11), PMID: 33658717
Paneth Cell	SI	Secrete ISC growth factors such as EGF and Wnt3 Secrete antimicrobial peptides, such as α-defensins	↓	↓	Eriguchi et al. (12), PMID: 22535662 Jenq et al. (13), PMID: 22547653 Hayase et al. (14), PMID: 29066578 Levine et al. (15), PMID: 23760615
Telocyte (at crypt base)	SI/LI	Secrete a ISC growth factor, R-Spondin 3	Unknown	Unknown	Shoshkes-Carmel et al. (16), PMID: 29720649
Tuft Cell	SI/LI	Stimulate ILC2 by production of IL-25	Unknown	Unknown	Gerbe et al. (17), PMID: 26762460 von Moltke et al. (18), PMID: 26675736

DCS cell, deep crypt secretory cell; ILC2, type 2 innate lymphoid cell; ILC3, type 3 innate lymphoid cell; ISC, intestinal stem cell; LEC, lymphatic endothelial cell; LI, large intestine; MRISC, Map3k2-regulated intestinal stromal cell; SI, small intestine.

a) Prolonged ILC2 reduction is induced by irradiation and/or chemotherapy. b) Reduction of ILC2 has been only demonstrated in the peripheral blood.

## Tissue Damages in GVHD

It has been recognized that bacterial and fungal pathogen-associated molecular patterns (PAMPs) such as lipopolysaccharide and α-mannan, play a critical role in initiating GVHD (20–23). PAMPs enhance production of proinflammatory cytokines, host alloantigen presentation, and infiltration of innate cells into the gastrointestinal tracts early after conditioning (21, 24, 25). Recent advances revealed the critical role of sterile damage-associated molecular patterns (DAMPs) in pathophysiology of GVHD. Tissue damage induced by conditioning chemotherapy and/or irradiation promotes release of DAMPs from damaged cells and initiates the inflammatory cascade which culminates in expansion of donor alloreactive T cells and development of acute GVHD. DAMPs are comprised of various molecules that are sequestrated in the cells in the steady state, while released into the extracellular space by cellular damages. Extracellular ATP activates host antigen presenting cells and inflammatory monocytes *via* the purinergic P2X7 and P2Y2 receptors, respectively, that exaggerates mouse GVHD (26, 27). It has been shown that lack of nucleotide-binding oligomerization domain–like (NOD) receptor protein 3 (NLRP3), a known target of ATP/P2X7 receptor signaling, in the recipient mice ameliorated GVHD, suggesting that ATP exaggerates GVHD *via* activation of NLRP3 inflammasome (28). ATP-induced NLRP3 activation in myeloid-derived suppressor cells reduces anti-GVHD effects of these cells after adoptive transfer (29). Another NLRP3 activator, uric acid is released into the extracellular space after conditioning and exaggerates GVHD (28). Interleukin-33 (IL-33) is released from epithelial cells after injury and promotes effector T-cell differentiation of donor T cells, that results in the exaggeration of GVHD (30, 31). Heparan sulfate and high-mobility group box 1 protein bind to toll like receptor 4 and induce GVHD after allo-SCT (32, 33).

DAMPs and PAMPs primarily activate myeloid inflammatory cells such as neutrophils and monocytes, and antigen presenting cells such as dendritic cells and macrophages. Conditioning-induced tissue damage promotes accumulation of host neutrophils and production of reactive oxygen species in the gastrointestinal tract, that in turn amplifies the tissue injury (25). Interestingly, neutrophils accumulated in the gastrointestinal tract early after conditioning migrate to mesenteric lymph nodes and promote activation of host antigen presenting cells and donor T cells (34). It has been shown that donor neutrophils also exaggerate GVHD (35). In patients’ samples, higher density of neutrophil infiltration in the gut was associated with worse outcomes of GVHD, further emphasizing critical role of neutrophils in pathophysiology of acute GVHD (36). Monocytes and inflammatory macrophages also contribute to development of GVHD by producing proinflammatory cytokines in response to DAMPs and PAMPs and promoting activation of donor T cells (23, 27, 37). Importantly, IL-12 produced from monocytes and macrophages after irradiation enhances antigen presentation by host non-hematopoietic cells and exaggerates GVHD (24). On the other hand, host tissue resident macrophage, the ontogenetically independent population from monocytes and inflammatory macrophages, plays a protective role against GVHD by suppressing donor T cell expansion (38–40).

## Tissue Stem Cells as Target of GvHD 

### Injury of ISCs in Intestinal GVHD

**Figure 1:**
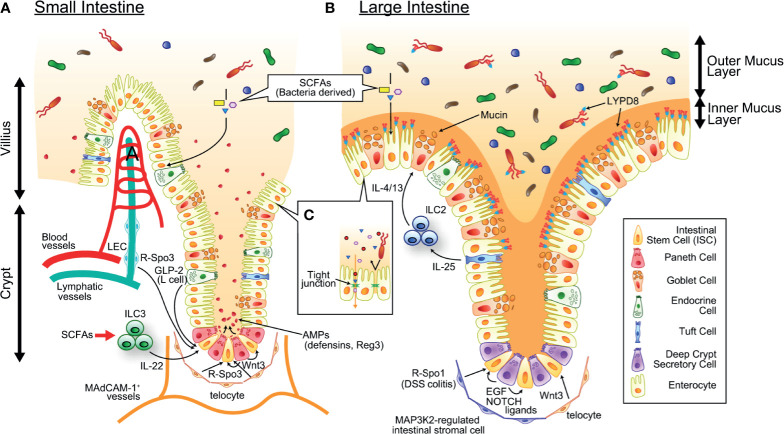
The mechanism maintaining intestinal homeostasis. ISCs residing at the crypt base give rise to all cell lineages in the epithelium and are supported by growth factors produced by definitive and putative niche components. SCFAs produced by commensal bacteria serves as energy source of intestinal epithelial cells. **(A)** In the small intestine, Paneth cells and telocytes produce Wnt3, telocytes and LECs produce R-Spo3, L cells produce GLP2, and ILC3s (green round cells in the figure) produce IL-22. Paneth cells also produce a large amount of AMPs such as α-defensins and REG3, and maintain healthy intestinal microbiota. **(B)** In the colon, deep crypt secretory cells produce EGF and NOTCH ligands, telocytes produce WNT3, and MAP3K2-regulated intestinal stromal cells produce R-Spo1. There are tremendous numbers of bacteria in the colonic lumen, which is segregated from epithelial cells by the inner mucus layer containing mucins produced by goblet cells and antimicrobial molecules such as REG3 and LYPD8 produced by enterocytes. IL-25 produced from Tuft cells stimulates ILC2s (blue round cells in figure) to secrete goblet cell growth factors such as IL-4 and IL-13. SCFAs produced by commensal bacteria serves as energy source of intestinal epithelial cells. **(C)** The intestinal epithelial tight junctions exhibit both size and charge selectivity and regulate the selective paracellular permeability, inhibiting penetration of bacteria and bacterial components while permitting the passage of water, ions, and small molecules. AMP, antimicrobial peptide; EGF, epithelial growth factor; GLP-2, Glucagon-like peptide 2; LEC, lymphatic endothelial cell; ILC2/3, type 2/3 innate lymphoid cell; IL-4/13/22/25, interleukin-4/13/22/25; ISC, intestinal stem cell; LYPD8, Ly6/PLAUR domain-containing protein 8; R-Spo1/3, R-spondin 1/3; SCFA, short-chain fatty acid.

Histological features of intestinal GVHD include epithelial apoptosis, crypt degeneration, and mucosal sloughing, as well as inflammatory cell infiltration (41). Early preclinical studies pointed out that proliferation of crypt cells was enhanced in less severe GVHD, while more severe GVHD abrogated crypt cell proliferation in association with villus atrophy and loss of the crypt; these findings indicated that severe GVHD targets putative tissue stem cells residing in the intestinal crypt (42). More recently, leucine-rich-repeat-containing G protein-coupled receptor 5 (LGR5) was found to be a unique marker for cycling intestinal stem cells (ISCs) residing at the crypt base of the small intestine and colon (43) (**Figure 1**). In the steady state, approximately 10 cells are produced every hour in each crypt and migrate to the villus tip in 2-3 days, and a lineage-tracing study using the LGR5-Cre reporter system revealed that LGR5^+^ ISCs give rise to all gut epithelial lineages (43, 44). Depletion of LGR5^+^ ISCs in the mice, in which diphtheria toxin receptor (DTR) was specifically expressed in LGR5^+^ cells, significantly delayed epithelial regeneration after irradiation-induced intestinal damage, suggesting that LGR5^+^ ISCs are important also for the regenerative process after gut injury (45). Adoptive transfer of eGFP-specific TCR-transgenic T cells (Jedi T cells) depleted LGR5-eGFP^+^ ISCs and profoundly impaired the regenerative response after irradiation, suggesting that ISCs are susceptible to T cell-mediated injury (46–48). Furthermore, the crypt base region is the primary site infiltrated by donor T cells after allo-SCT; donor T cells migrate to the crypt base region in a MAdCAM-1-dependent manner as early as day 4 after murine allo-SCT, suggesting that ISCs are the primary target of gut GVHD (49).

**Figure 2:**
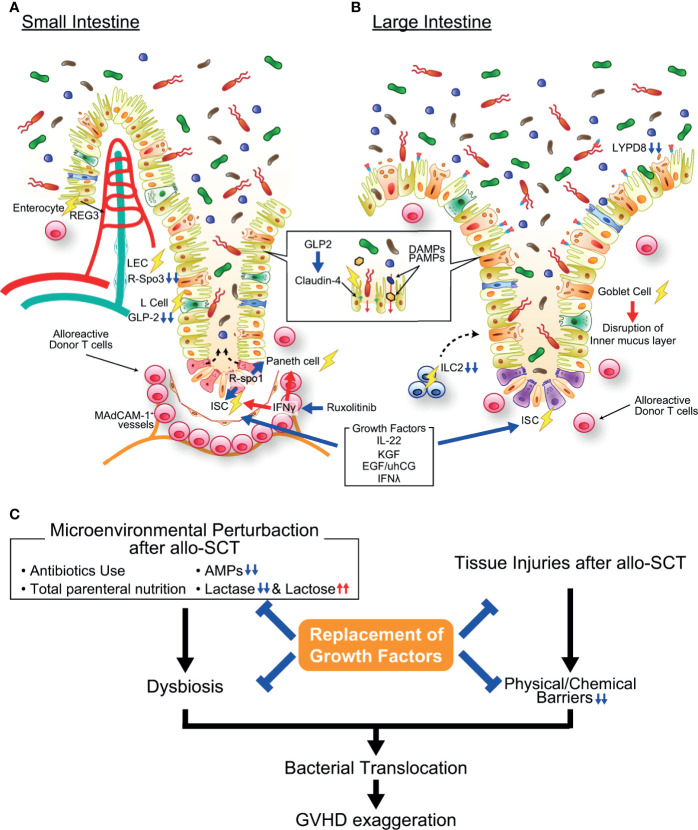
Pathophysiology of gastrointestinal graft-versus-host disease (GVHD). **(A)** In the small intestine, activated alloreactive donor T cells (pink round cells in figure) migrate to the crypt base region early after allogeneic transplantation in a MAdCAM-1-dependent manner and damage ISCs, resulting in impairment of mature intestinal epithelial cell regeneration. Paneth cell injury causes the reduction of AMP production and loss of function as an ISC niche. IFN-γ plays an important role in both ISC and Paneth cell injury in GVHD, and ruxolitinib protects ISCs and Paneth cells against GVHD. Moreover, growth factors of ISCs such as R-Spondin 3, IL-22, and GLP-2 are reduced in the intestine due to GVHD-induced reduction of LECs, ILC3s, and L cells. The expression of tight junction molecules such as claudin-4 are also reduced in GVHD, resulting in disruption of intestinal epithelial barrier function. **(B)** In the large intestine, goblet cell injury in GVHD results in disruption of the mucus layers bleaching both chemical and mechanical barrier functions of the intestinal mucosa. ILC2s, producer of goblet cell growth factors, are profoundly depleted by conditioning radiotherapy or chemotherapy, likely inhibiting regeneration of goblet cells. **(C)** Microenvironmental perturbation after allo-SCT induced by administration of antibiotics and/or total parenteral nutrition, reduction of AMP production, and lactose malabsorption leads to intestinal dysbiosis, frequently accompanying *Enterococcus* domination. Dysbiosis and disruption of barrier function of the intestinal mucosa enhance bacterial translocation, further exaggerating GVHD. Replacement of growth factors for ISCs, Paneth cells, and goblet cells ameliorate GVHD. Allo-SCT, allogeneic hematopoietic stem cell transplantation; DAMP, damage-associated molecular pattern; EGF, epidermal growth factor; IFN-γ, interferon-γ; KGF, keratinocyte growth factor; LYPD8, Ly6/PLAUR domain-containing protein 8; PAMP, pathogen-associated molecular pattern; REG, regenerating islet-derived protein; R-Spo1, R-spondin1; uhCG, urinary-derived human chorionic gonadotropin.

A landmark study by Takashima et al. demonstrated that ISCs marked by another ISC-specific marker, olfactomedin-4 (Olfm4), are targeted by intestinal GVHD (7) (**Figure 2**). The reduction of LGR5^+^ ISCs in intestinal GVHD was then confirmed using a LGR5 reporter system (5). Due to the rapid turnover of gut epithelial cells, depletion of cycling ISCs in the crypt in intestinal GVHD soon leads to villus atrophy and causes refractory colitis (44). In the small intestine, quiescent Bmi1^+^ stem cells exist at four cell diameters above the base of the crypt and are called +4 stem cells (50). These cells are activated only after severe gut injury or depletion of LGR5^+^ ISCs and differentiate into all types of epithelial cells, including LGR5^+^ ISCs (51, 52). However, the fate and role of this second stem cell population in GVHD remain to be clarified. The mechanisms by which GVHD causes injury of LGR5^+^ ISCs have been studied intensively using a gut organoid culture system. Single LGR5^+^ ISCs isolated from the intestine give rise to crypt–villus organoids containing all differentiated cell types of the intestinal epithelium without the support of niche cells (53). Coculture of intestinal organoids with activated T cells induced caspase-3/caspase-7 cleavage and apoptosis of LGR5^+^ ISCs in the organoid, while IFN-γ blockade prevented T cell-mediated injury of the organoids, indicating that activated T cells damage LGR5^+^ ISCs in an IFN-γ-dependent manner (8, 54). In mouse models of allo-SCT, significantly more LGR5^+^ ISCs persisted after transplantation with IFN-γ-deficient donor T cells than after transplantation with wild-type (WT) donor T cells (54). Furthermore, administration of IFN-γ significantly reduced LGR5^+^ ISCs in the mice conditioned with total body irradiation, while it induced a proliferative response in the crypt in nonirradiated mice (54). Thus, IFN-γ seems to be more harmful for ISCs in the presence of genotoxic stress, such as irradiation, *in vivo*, while a high concentration of IFN-γ alone could induce apoptosis of ISCs *in vitro*. Alternatively, radiosensitive niche components could protect ISCs from IFN-γ *in vivo* to some extent.

### Injury of the ISC Niche

The ISC niche, which provides survival and growth factors for ISCs, is also targeted by GVHD (**Figures 1** and **2**). Interleukin-22 (IL-22) produced by type 3 innate lymphoid cells (ILC3s) is a well-described growth factor of LGR5^+^ ISCs (5). Total body irradiation (TBI) enhances IL-22 production from radioresistant ILC3s in an interleukin-23 (IL-23)-dependent manner, which is believed to promote regeneration of epithelial cells from radiation-induced damage. IL-22-producing ILC3s persisted after syngeneic bone marrow transplantation, while they were depleted after mouse allogeneic transplant, indicating that GVHD targets ILC3s. A reduction in IL-22 producing ILC3s in GVHD is associated with prolonged depletion of ISCs and exacerbation of gut GVHD.

Crypt bases have enriched transcription of Wnt target genes, and Paneth cells produce high levels of Wnt3, suggesting that Paneth cells are an ISC niche component (55, 56). Although the survival and proliferation of LGR5^+^ ISCs were not affected in Paneth cell-deficient mice in the steady state, Paneth cells may protect ISCs against gut injury (57). Because Paneth cells are also susceptible to IFN-γ-induced apoptosis in GVHD, regeneration of the gut epithelium from ISCs could be further disturbed in intestinal GVHD (12, 13). On the other hand, Paneth cell-derived Wnt3 is redundant with that produced from subepithelial telocytes (16, 58–60). It remains to be clarified whether telocytes are targeted by GVHD. In the colon, which is devoid of Paneth cells, deep crypt secretory (DCS) cells residing at the crypt base act as the niche for LGR5^+^ ISCs by producing NOTCH ligands and epidermal growth factor (EGF) (1). While DCS cells do not produce Wnt ligands, stromal tissues surrounding colonic crypts produce Wnt ligands and support colonic ISCs (58). It also remains to be clarified whether DCS cell are targeted by GVHD. R-spondins are the ligands of LGR4, LGR5, and LGR6 and enhance Wnt/β-catenin signaling by preventing ubiquitination and degradation of the Wnt receptor Frizzled (61, 62). The R-spondin family is composed of four molecules. R-Spo1-R-Spo4 share a similar structure, and each of these four molecules can bind to LGR4, LGR5 and LGR6 (63). We found that R-Spo3 is the major molecule produced in the small intestine (10). Although it has been reported that mesenchymal cells, including telocytes, produce R-Spo3, we found that CD90^+^CD31^+^podoplanin^+^ lymphatic endothelial cells are the main producers of R-Spo3 in the intestine (10, 16). Importantly, both the R-Spo3 production and absolute numbers of lymphatic endothelial cells are significantly reduced in GVHD (10). On the other hand, it remains to be clarified whether R-Spo3-producing telocytes are targeted by GVHD. The importance of R-Spo3 was also demonstrated in an antibody-mediated inhibition study, in which administration of anti-R-Spo3 antibodies alone reduced LGR5^+^ ISCs in naïve mice and suppressed the regenerative response after irradiation (64). Although this study showed that anti-R-Spo2 antibodies and anti-R-Spo3 antibodies work synergistically in the depletion of LGR5^+^ ISCs, the cellular source of R-Spo2 in the intestine remains to be clarified. Interestingly, recent study showed that Map3k2-regulated intestinal stromal cells (MRISCs) residing around the crypt base enhance production of R-Spo1 in response to dextran sodium sulfate (DSS)-induced colitis and protect colonic ISCs (11). Map3k2-deficient mice are more susceptible to DSS-induced colitis compared with wild type controls, further emphasizing a protective role of MRISCs against inflammation of the colon (11). These findings suggest that there are distinct ISC niche systems in the small intestine and the colon, and further studies are required to assess the fate of these ISC niches in GVHD.

### GVHD Prophylaxis and Treatments Targeting ISCs

Strategies that protect ISCs or induce their regeneration could be therapeutic options for GVHD that avoid strengthening immune suppression, which could lead to infection or leukemia relapse. As mentioned above, the reduction in IL-22 produced by ILC3s in GVHD leads to depletion of ISCs. IL-22 induces the proliferation and differentiation of ISCs and inhibits the apoptosis of ISCs after genotoxic stress (65). Replacement of IL-22 by administration of F-652, a recombinant fusion protein consisting of an rhIL-22 dimer and Fc fusion protein, after mouse allogeneic bone marrow transplantation enhanced the recovery of ISCs, increased epithelial regeneration, and ameliorated GVHD (6). However, the potential benefit of IL-22 could be limited because IL-22 secreted from donor T cells has been shown to aggravate GVHD by reducing Tregs and enhancing inflammatory responses (66–68). It has been suggested that IL-22 induces Th1 cell infiltration in the gastrointestinal tract *via* a host type I interferon dependent manner (69). Thus, the safety and efficacy of IL-22 replacement therapy must be evaluated in clinical studies; F-652 is now being tested for the treatment of lower gastrointestinal acute GVHD (NCT02406651). Because ILC3s produce IL-22 in response to bacterial metabolites such as short-chain fatty acids (SCFAs), probiotics that produce SCFAs could be used for GVHD prophylaxis (70).

Administration of R-spondins is also promising for GVHD prophylaxis, as this strategy protects ISCs against mouse GVHD. Recombinant human R-Spo1 (rhR-Spo1) was found to stimulate the proliferation of epithelial cells in the intestinal crypt (71). Subsequently, it was shown that rhR-Spo1 expands ISCs in naïve mice and mice undergoing allo-SCT. Importantly, rhR-Spo1 administered in the peritransplant period protects ISCs against GVHD and ameliorated GVHD after allo-SCT in TBI-conditioned mice (7). In contrast, rhR-Spo1 does not impact the severity of GVHD after allo-SCT without conditioning, potentially indicating synergistic effects of TBI and T-cell-derived IFN-γ on ILC injury (7, 54). Administration of a Robo ligand, Slit2 works synergistically with R-Spo1 in preventing ISC loss after chemoradiotherapy, suggesting that this combination could be useful for GVHD prophylaxis (72).

Type III interferon plays a protective role against gastrointestinal GVHD. Type III interferon family was discovered in 2003 and consists of four molecules, IFN-λ1 (IL-29), IFN-λ2 (IL-28A), IFN-λ3 (IL-28B), and IFN-λ4 (73). Among them, IFN-λ2 and IFN-λ3 are expressed in both humans and mice, while IFN-λ1 gene is a pseudogene in mice, and IFN-λ4 gene is absent in mice. IFN-λ receptor consists of two chains, including a unique subunit, IFN-λ receptor 1 (IFNLR1) and common IL-10 receptor-β (IL-10RB) chain, which is shared with cytokines of the IL-10 family. IFNLR1 is preferentially expressed in gastrointestinal epithelium, suggesting that IFNλ is a key effector cytokine in mucosal immunity (74, 75). Recently, Henden and colleagues showed that IFNλ treatment improves the proliferative and regenerative capacity of LGR5^+^ ISCs independently of IL-22 and ameliorates murine GVHD (76). Since, pegylated recombinant IL-29 is being developed as an adjunctive therapy for Hepatitis C, this agent may be rapidly testable for clinical GVHD (77).

Ruxolitinib, a JAK1/2 inhibitor, has been shown to ameliorate mouse and human GVHD and has been approved by the Food and Drug Administration (FDA) in the United States for the treatment of steroid-refractory acute GVHD (78, 79). Ruxolitinib profoundly suppresses T cell activation, proliferation, and differentiation toward T helper 1 (Th1), Th17 and cytotoxic T cells (79). Given the critical role of IFN-γ in ISC injury, it has been tested if ruxolitinib could protect ISCs against GVHD by inhibiting JAK1/2-STAT1 pathway, an indispensable pathway in IFN-γ receptor signaling. Organoid culture systems have demonstrated that allogeneic T cells induce apoptosis of organoids and ISCs in an IFN-γ-dependent manner (8). Ruxolitinib protected ISCs and Paneth cells in organoids from IFN-γ and allogeneic T cells (8, 54). Furthermore, ruxolitinib prevented IFN-γ-induced ISC injury after syngeneic SCT, indicating that ruxolitinib protects ISCs independent of suppression of allogeneic T cell activation (54). These ISC-targeting strategies for GVHD prophylaxis and treatment are promising and could promote regeneration of all types of intestinal epithelial cells after GVHD-mediated injury (**Figure 2**).

### Tissue Stem Cells in Other Organs

Tissue stem cells in other target organs, such as the skin and liver, could be involved in GVHD pathophysiology. The fate of skin stem cells in acute cutaneous GVHD has been studied. Multiple tissue stem and/or progenitor populations of epithelial cells have been identified in the skin. The bulge of hair follicles has long been recognized to foster tissue stem cells because long-lived label-retaining cells exist in the hair bulge (80). More recently, it became possible to identify hair follicle stem cells (HFSCs) in the lower part of the bulge as CD34^+^, cytokeratin 15 (CK15)^+^, and LGR5^+^ cells using flow cytometric or immunofluorescent studies (81–83). These HFSCs alone can regenerate all structures of hair follicles and hair shafts and contribute to regeneration of the epidermis after skin injury (19, 82, 84). In addition to HFSCs, LGR6^+^ stem cells residing directly above the bulge and leucine-rich repeats and immunoglobulin-like domains 1 (Lrig1)^+^ stem cells in the isthmus maintain the upper pilosebaceous units (85, 86). Other than stem cells in the hair follicles, there are CK15^+^ epidermal progenitor and/or stem cells in the rete-like prominences (RLPs) of mouse tongues, a surrogate of human epidermal rete ridges of the skin.

Early studies demonstrated that donor T cells primarily migrate to stem cell-rich parts of the skin, such as mouse RLPs, human rete ridges, and the bulge of hair follicles, suggesting that skin stem cells could be targeted by GVHD (87–90). Among multiple stem cell populations, CK15^+^ stem and/or progenitor cells in mouse RLPs have been shown to undergo cytokine-induced apoptosis in cutaneous GVHD (90–92). Recently, we found that LGR5^+^ HFSCs were significantly reduced in mouse cutaneous GVHD, in association with reduced numbers of hair follicles, alopecia, and delayed wound healing (19). This finding was rather surprising because a previous study showed that injection of eGFP-specific Jedi T cells did not deplete LGR5-eGFP^+^ HFSCs, suggesting that these LGR5^+^ HFSCs are immune privileged (47). This discrepancy suggests that HFSCs are not inherently immune privileged and that the environment and/or HFSC niche protect HFSCs against immune-mediated injury. An extensive inflammatory environment or disruption of the HFSC niche could be responsible for HFSC damage in cutaneous GVHD. One of the HFSC niche components, subcutaneous fat, which acts as regulator of hair cycling and energy reservoir for HFSCs, becomes atrophic in cutaneous GVHD, which can lead to a reduction of LGR5^+^ HFSCs (19, 93–95). Although the mechanism by which GVHD depletes LGR5^+^ HFSCs and CK15^+^ RLP stem cells remains to be clarified, it is worth of note that topical administration of ruxolitinib protects these stem cells from mouse GVHD (19). On the other hand, topical corticosteroids demonstrate direct toxicity that leads to depletion of HFSCs after syngeneic and allogeneic SCT, even though topical steroids dramatically reduce donor T cell infiltration to the skin in cutaneous GVHD. Protection of LGR5^+^ HFSCs with topical ruxolitinib was associated with suppression of alopecia and enhancement of wound healing after allo-SCT, while topical corticosteroid was not (19). Based on its protective effects on both ISCs and skin stem cells, ruxolitinib could be an ideal therapeutic agent for GVHD (78, 79). The fate of other stem cell populations in the skin, such as LGR6^+^ stem cells and Lrig1^+^ stem cells in the hair follicles, remains to be clarified (85, 86).

The liver, another major target organ in acute GVHD, is a highly regenerative organ, and there are two main epithelial populations: hepatocytes and biliary epithelial cells (BECs). Lineage-tracing studies have shown that there are stem and/or progenitor populations of hepatocytes that maintain the hepatocyte pool in steady states, for example, studies in Axin2-Cre/ER reporter mice (96). However, some of these lineage-tracing strains have aberrant proliferation of labeled hepatocytes, possibly due to deletion of exons of the target molecule, which potentially leads to overestimation of the contribution of labeled cells to tissue regeneration after liver injury (97). More recently, it has been shown that mid-lobular hepatocytes cycle and maintain whole hepatocytes in the liver, except glutamine synthetase (GS)-expressing hepatocytes facing the central vein, which are maintained independently from other hepatocytes (97, 98). In certain contexts of liver injury, LGR5^+^ and Sox9^+^ hepatocytes endowed with the potential to differentiate both into hepatocytes and BECs emerge, and BECs proliferate and contribute to the reconstitution of hepatocytes after severe liver injury (99–103). Because jaundice and biliary dysfunction are the cardinal features of liver GVHD, it should be more important to study BEC stem cells rather than stem cells of hepatocytes. Huch et al. found that single LGR5^+^ cells isolated from the hepatic duct give rise to liver organoids that can be differentiated into both hepatocytes and BECs (99). The fate of BEC stem cells needs to be clarified in future studies.

## Alteration of the Microenvironment Induces Intestinal Dysbiosis in GVHD

Intestinal dysbiosis is frequently observed after allo-SCT and is associated with exacerbation of GVHD and transplantation-related death (13, 104, 105). Multiple factors, such as antibiotics and total parenteral nutrition, can lead to dysbiosis after allo-SCT (**Figure 2** and **Table 1**). In addition, GVHD-induced tissue injury can generate a microenvironment related to dysbiosis. α-Defensins, major antimicrobial peptides (AMPs) produced from Paneth cells, exert potent bactericidal effects on pathogenic bacteria that occupy a minor proportion of the healthy microbiota but are minimally effective on nonpathogenic commensals that dominate the healthy gut microbiota (106, 107). Paneth cells are highly sensitive to GVHD, and α-defensin production is profoundly decreased in GVHD (12, 13, 108). This reduction is mediated by IFN-γ signaling, and ruxolitinib can protect Paneth cells against GVHD (54). R-Spo1, a growth factor of ISCs, is also a potent inducer of Paneth cell differentiation from ISCs, and we found that administration of rhR-Spo1 induced expansion of Paneth cells in naïve mice, leading to marked elevation of fecal levels of α-defensins such as cryptdin-1 (Crp-1) and cryptdin-4 (Crp-4) (14). In mouse GVHD, peritransplant administration of R-Spo1 protects not only ISCs but also Paneth cells, resulting in preserved α-defensin production and prevention of intestinal dysbiosis after allo-SCT (14). Short-term oral administration of Crp-4 to allogeneic recipient mice temporally mitigated intestinal dysbiosis and inflammation in the gut after allo-SCT, while dysbiosis developed after cessation of Crp-4 treatment, indicating that long-term administration of Crp-4, until Paneth cell regeneration, is required for the prevention of dysbiosis after allo-SCT (14). Paneth cell numbers in duodenal biopsies from transplanted patients are negatively related to gut GVHD severity, further emphasizing the protective role of Paneth cells against GVHD (15).

REG3, another major AMP in the intestine, is produced by intestinal epithelial cells, including Paneth cells and enterocytes, and diffuses into the inner mucus layer, segregating luminal bacteria from the gut epithelium (109, 110). In mouse models of allo-SCT, the expression levels of REG3γ, the mouse homolog of human REG3α, in the small intestine were significantly reduced in GVHD, and REG3γ leaked from the gut to the blood, leading to elevation of plasma levels of REG3γ (111, 112). In clinical allo-SCT, the plasma levels of REG3α and ST2 are now widely appreciated as diagnostic and prognostic biomarkers of acute GVHD (113–115). In mouse models of steroid-refractory GVHD, it has been shown that IL-22 produced by donor Th/Tc22 cells stimulates REG3γ production in the intestine, and excess REG3γ leads to dysbiosis and exacerbation of GVHD (67). Thus, REG3γ could be a therapeutic target for treating steroid-refractory GVHD.

*Enterococcus* domination, defined as a status in which 30% or more of all the bacteria in the fecal microbiota are enterococci, develops frequently after allo-SCT and is associated with blood stream infection, development and exacerbation of GVHD, and GVHD-related death after allo-SCT (105, 116–119). Because the presence of the *VanA* gene in fecal samples from allo-SCT recipients has been associated with *Enterococcus* domination, antibiotics likely contribute to the development of *Enterococcus* domination (119). However, *Enterococcus* domination is also observed after murine allo-SCT in which no antibiotics are used, indicating that antibiotics are not the only reason for *Enterococcus* domination and that GVHD may induce a microenvironment suitable for the expansion of enterococci. The growth of enterococci is strictly dependent on lactose, and the expression of lactase, a critical enzyme for the absorption of lactose from the diet, in the intestine is reduced in GVHD (119, 120). The reduction in lactase in GVHD leads to ineffective absorption and an increase in lactose availability in the gut lumen, leading to enterococcal expansion. Importantly, the lactose intolerance allele is associated with the persistence of *Enterococcus* domination after the cessation of antibiotics. These data suggest that a lactose-free diet or lactase administration could be used for prophylactic treatment of *Enterococcus* domination, which could improve the outcomes of allo-SCT. In addition to antibiotic administration and lactase reduction, reduction of α-defensins, which exert potent bactericidal effects on *Enterococcus*, could contribute to enterococcal expansion after allo-SCT (107).

## The Barrier Function of Gut Epithelial Cells in GVHD

The intestinal mucosa has the complex task of acting as a semipermeable barrier that allows the absorption of nutrients and water while limiting the transport of potentially harmful microbes and microbial components. Sheets of gut epithelial cells are bound to each other *via* tight junctions, acting as a physical barrier against luminal components (**Figure 1**). Conditioning and allogeneic T cell responses damage epithelial cells (**Table 1**), leading to the loss of the physical barrier function of the mucosal epithelium against bacteria and bacterial components , which fosters an environment prone to GVHD development (121). Thus, epithelial growth factors have been proposed as therapeutic options for acute GVHD (**Figure 2**). Keratinocyte growth factor (KGF) promotes the proliferation and differentiation of epithelial cells. It was suggested that ISCs are also supported by KGF; however, whether KGF protects ISCs against GVHD has not been explored using specific ISC markers, such as LGR5 (122, 123). Although it has been reported that KGF ameliorates murine gut GVHD (124, 125), human recombinant KGF did not demonstrate significant beneficial effects on the incidence and severity of GVHD in randomized clinical trials (126–128). Although the reason for this discrepancy between preclinical and clinical studies is not fully understood, it has been suggested that KGF could exert more potent anti-GVHD effects in recipients conditioned with TBI alone than in those conditioned with TBI in combination with cytotoxic agents; the latter strategy is used in the clinical setting (126).

Glucagon-like peptide 2 (GLP-2) is another growth factor of gut epithelial cells, and administration of a GLP-2 analog protects ISCs against irradiation-induced injury (129). GLP-2-producing enteroendocrine L cells are targeted by GVHD, and reduction of L cells in the patients’ colon is associated with worse outcome after allo-SCT. Because GLP-2 is inactivated by DPP-4, a DPP-4-resistant GLP-2 analog, teduglutide, was tested for a GVHD prophylaxis. Peritransplant administration of teduglutide protected ISCs and Paneth cells against GVHD, and prolonged survival after mouse allo-SCT (9). Furthermore, GLP-2 and GLP-2 analogues enhance the expression of tight junction molecules such as claudin-4, possibly enhancing intestinal barrier function in GVHD (9, 130). A clinical trial in which teduglutide is tested for treatment of short bowel syndrome (NCT04733066) is ongoing, and future clinical studies are required to test if teduglutide could protect patients against GVHD. Interestingly, a small-scale phase II study demonstrated that peritransplant administration of high-dose sitagliptin, a DPP-4 inhibitor, prevented the onset of acute GVHD (131). Although it is most likely that DPP-4 inhibition prevents GVHD by suppressing donor T cell activation (132), DPP-4 inhibition may mitigate damage to the intestinal epithelium by inhibiting GLP-2 degradation (133). The impact of DPP-4 inhibitors on GVHD-induced damage to gut epithelial cells needs to be clarified in future studies.

In rodent models of radiation colitis, administration of EGF enhanced gut epithelial regeneration (134, 135). In a phase I clinical trial, it has been shown that administration of a urinary-derived human chorionic gonadotropin (uhCG) agent containing abundant EGF was safe and possibly effective for the treatment of high-risk or steroid-refractory acute GVHD (136). This agent may improve GVHD *via* EGF-induced protection of gut epithelial cells, while the Treg expansion observed after administration of this agent could contribute to GVHD suppression, too. This inexpensive and commercially available uhCG agent will be studied in phase II and III trials.

In mouse GVHD, the TNF-α/MLCK210 axis increases tight junction permeability to larger molecules (137). IFN-γ also regulates tight junction permeability (138). Thus, GVHD actively increases permeability through tight junctions, which promotes the absorption of bacterial components, further recruiting donor T cells and propagating GVHD (137). Prevention of increase of tight junction permeability could be another prophylactic strategy against GVHD.

Loss of commensals in intestinal microbiota after allo-SCT leads to the reduction of bacterial metabolites which contribute to maintenance of tissue homeostasis. Among these metabolites, butyrate is mainly produced by commensal anaerobes such as *Clostridia* and *Blautia*, and mitigates harmful immune reactions by promoting differentiation of regulatory T cells (139). Microbiota-derived butyrate is also taken up by intestinal epithelial cells through G-protein coupled receptor, GPR43 and serves as a major energy source of intestinal epithelial cells. Butyrate acts as a histone deacetylase (HDAC) inhibitor and promotes tricarboxylic acid cycling, improving integrity of barrier function of intestinal mucosa (140, 141). Thus, dysbiosis with the reduction of butyrogenic bacteria reduces butyrate in the intestinal epithelial cells and impairs the resilience of the gut epithelium after allo-SCT (142). Probiotics containing butyrogenic bacteria or prebiotics containing butyrogenic fibers and starch are promising therapeutic options against mouse and human GVHD (140, 143). The urinary levels of 3-indoxyl sulfate (3-IS) are positively correlated with the abundances of *Lachnospiraceae* and *Ruminococcaceae* in the gut microbiota, and higher levels of urinary 3-IS predicts better survival after allo-SCT. Although the direct role of 3-IS in GVHD remains to be clarified, 3-IS could act as a ligand for aryl hydrocarbon receptor, the critical receptor for maintenance of intestinal epithelial barrier function and production of AMPs (144–146).

## The Role of the Gut Mucus Layer in GVHD

The intestinal mucus layer constitutes a critical barrier that segregates millions of microbes and environmental antigens in the gut lumen from the host immune system (**Figure 1**). The mucus layer serves as the first line of innate defense, and gel-forming mucins secreted by goblet cells form the basic scaffold of the mucus layer. Mice lacking the *Muc2* gene, encoding the major gel-forming mucin in the intestine, are devoid of mucus layers and prone to developing severe colitis, suggesting that direct contact between luminal bacteria and the intestinal mucosa triggers inflammation (147, 148). The large intestine has a system with two mucus layers; the inner mucus layer is enriched with antimicrobial molecules (AMMs), such as Ly6/Plaur domain-containing 8 (LYPD8), and devoid of bacteria, suggesting that the mucus layer also acts as a chemical barrier against luminal bacteria (149, 150).

As noted above, GVHD-induced Paneth cell injury and lactose malabsorption together with other factors, such as antibiotic administration and induction of total parenteral nutrition, lead to intestinal dysbiosis after allo-SCT. In such a situation, the mucosal barrier must act as the final line of defense against pathogenic bacteria expanding in the gut lumen. However, GVHD leads to a reduction in intestinal goblet cells, which results in disruption of the mucus layer due to its rapid turnover (**Figure 2** and **Table 1**); the mucus layer is renewed every 1 to 2 hours by newly produced mucus from goblet cells (151–153). Recently, we studied the role of goblet cells and the inner mucus layer in the pathophysiology of acute GVHD using mouse models of acute GVHD (2). First, we confirmed that goblet cells were profoundly and persistently reduced in the colon after allo-SCT, which led to disruption of the colonic two-layered mucus system in allogeneic recipients in association with enhanced bacterial translocation, elevated plasma levels of proinflammatory cytokines, and exacerbation of GVHD. Although the mechanism by which GVHD targets goblet cells remains to be clarified, it is possible that goblet cells are reduced due to GVHD-induced depletion of ISCs considering the rapid turnover of goblet cells (3 to 7 days) (154). In the steady state and after parasite infections, ILC2s produce growth factors of goblet cells, such as interleukin 13 (IL-13), in response to interleukin-25 (IL-25) secreted from Tuft cells (17, 18). We found that pretransplant administration of IL-25 expanded goblet cells that persisted after GVHD, preventing bacterial translocation, elevation of proinflammatory cytokines, and exacerbation of GVHD (2). Conditioning TBI and chemotherapy lead to prolonged depletion of ILC2s in mice and humans (3, 4), which could further reduce goblet cells or impair regeneration of these cells. Bruce et al. showed that donor ILC2 infusion promotes IL-13 production by ILC2s and enhances the survival of donor myeloid suppressor cells, suppresses donor T cell production of proinflammatory cytokines, and reduces GVHD (3). Although this study demonstrated that transfer of donor ILC2s improves intestinal epithelial integrity, the impact on goblet cells was not addressed. Deficiency of NOD-like receptor family pyrin domain-containing 6 (NLRP6), the critical molecule for goblet cell secretion of mucus, in nonhematopoietic cells of recipients mitigates goblet cell injury after allo-SCT and ameliorates intestinal GVHD, suggesting that NLRP6 is another target molecule for protection of goblet cells after allo-SCT (155).

LYPD8 is produced by enterocytes in the colon and enriched in the inner mucus layer (149). LYPD8 binds to flagellated bacteria such as *Escherichia coli* and prevents bacterial translocation by inhibiting bacterial motility. Based on these findings, we studied the protective role of LYPD8 in murine GVHD using LYPD8-deficient mice as recipients (2). First, we found that disruption of the inner mucus layer in allogeneic recipients led to disappearance of the LYPD8-rich layer in the mucus layer. Next, we found that bacterial translocation was dramatically enhanced in LYPD8-deficient recipients compared to WT recipients after allo-SCT, in association with exacerbation of GVHD. Furthermore, goblet cell expansion using IL-25 did not ameliorate GVHD in LYPD8-deficient recipients, suggesting that the mucus layer containing LYPD8 is critical for goblet cell-mediated GVHD suppression (2).

## Concluding Remarks

The discovery of specific markers for tissue stem cells has enabled us to study the fate of tissue stem cells in mouse GVHD, and we found that ISCs and HFSCs are targeted by GVHD. Furthermore, niche components that support tissue stem cells are also damaged after allo-SCT, likely inhibiting the recovery of tissue stem cells after GVHD-mediated injury. Emerging evidence also indicates that human and mouse GVHD targets specific epithelial populations, such as Paneth cells, L cells, and goblet cells, resulting in disruption of tissue homeostasis (**Figure 2** and **Table 1**). Strategies to promote recovery of tissue stem cells and maintenance of the tissue microenvironment are promising adjuncts to standard immunosuppressive GVHD prophylaxis and treatment, which may enable the separation of GVHD and graft-versus-leukemia effects.

There remain many unanswered questions in this field. Although the existence of LGR5^+^ ISCs is also demonstrated in the human intestine, the fate of LGR5^+^ tissue stem cells in the intestine and skin after human allo-SCT remains to be clarified (156). The role and fate of BEC stem cells need to be studied both in human and mouse liver GVHD. Furthermore, the role of tissue stem cells in pathophysiology of chronic GVHD has not been well studied, and studies about intestinal dysbiosis in chronic GVHD has only just begun (157). Although it has been shown that protection of intestinal stem cells, Paneth cells, or goblet cells represents a promising anti-GVHD treatment, these strategies have been tested only in mouse models of GVHD in a prophylactic manner. It should be tested if these strategies are also useful for treatment of established GVHD.

## Author Contributions

All authors contributed to the article and approved the submitted version.

## Funding

This study was supported by JSPS KAKENHI (21K16259 to TA, 21K08409 to DH).

## Conflict of Interest

The authors declare that the research was conducted in the absence of any commercial or financial relationships that could be construed as a potential conflict of interest.

## Publisher’s Note

All claims expressed in this article are solely those of the authors and do not necessarily represent those of their affiliated organizations, or those of the publisher, the editors and the reviewers. Any product that may be evaluated in this article, or claim that may be made by its manufacturer, is not guaranteed or endorsed by the publisher.
